# Skeletal muscle mass correlates with increased toxicity during neoadjuvant radiochemotherapy in locally advanced esophageal cancer: A SAKK 75/08 substudy

**DOI:** 10.1186/s13014-019-1372-3

**Published:** 2019-09-11

**Authors:** Cédric M. Panje, Laura Höng, Stefanie Hayoz, Vickie E. Baracos, Evelyn Herrmann, Helena Garcia Schüler, Urs R. Meier, Guido Henke, Sabina Schacher, Hanne Hawle, Marie-Aline Gérard, Thomas Ruhstaller, Ludwig Plasswilm, for the Swiss Group for Clinical Cancer Research (SAKK)

**Affiliations:** 1 Department of Radiation Oncology, Kantonsspital St. Gallen, St. Gallen, Switzerland; 2 Department of Radiation Oncology, Luzerner Kantonsspital, Luzern, Switzerland; 3 Swiss Group for Clinical Cancer Research (SAKK) Coordinating Center, Bern, Switzerland; 4 Division of Palliative Care Medicine, Department of Oncology, University of Alberta, Edmonton, Alberta Canada; 5 Department of Radiation Oncology, Inselspital Bern, Bern, Switzerland; 6 University Hospital Zurich, Zurich, Switzerland; 7 Department of Radiation Oncology, Kantonsspital Winterthur, Winterthur, Switzerland; 8 Department of Medical Oncology, Kantonsspital Winterthur, Winterthur, Switzerland; 9 Department of Medical Oncology and Hematology, Kantonsspital St. Gallen and University of Basel, Basel, Switzerland; 10 Department of Radiation Oncology, Kantonsspital St Gallen, St Gallen and University of Bern, Bern, Switzerland

**Keywords:** Sarcopenia, Esophageal cancer, Resectable, Locally advanced, Radiotherapy, Radiochemotherapy, Cetuximab

## Abstract

**Background:**

Sarcopenia, the critical depletion of skeletal muscle mass, is an independent prognostic factor in several tumor entities for treatment-related toxicity and survival. In esophageal cancer, there have been conflicting results regarding the value of sarcopenia as prognostic factor, which may be attributed to the heterogeneous patient populations and the retrospective nature of previous studies. The aim of our study was therefore to determine the impact of sarcopenia on prospectively collected specific outcomes in a subgroup of patients treated within the phase III study SAKK 75/08 with trimodality therapy (induction chemotherapy, radiochemotherapy and surgery) for locally advanced esophageal cancer.

**Methods:**

Sarcopenia was assessed by skeletal muscle index at the 3rd lumbar vertebra (L3) in cross-sectional computed tomography scans before induction chemotherapy, before radiochemotherapy and after neoadjuvant therapy in a subgroup of 61 patients from four centers in Switzerland. Sarcopenia was determined by previously established cut-off values (Martin et al., PMID: 23530101) and correlated with prospectively collected outcomes including treatment-related toxicity, postoperative morbidity, treatment feasibility and survival.

**Results:**

Using the published cut-off values, the prevalence of sarcopenia increased from 29.5% before treatment to 63.9% during neoadjuvant therapy (*p* < 0.001). Feasibility of neoadjuvant therapy and surgery was not different in initially sarcopenic and non-sarcopenic patients. We observed in sarcopenic patients significantly increased grade ≥ 3 toxicities during chemoradiation (83.3% vs 52.4%, *p* = 0.04) and a non-significant trend towards increased postoperative complications (66.7% vs 42.9%, *p* = 0.16). No difference in survival according to sarcopenia could be observed in this small study population.

**Conclusions:**

Trimodality therapy in locally advanced esophageal cancer is feasible in selected patients with sarcopenia. Neoadjuvant chemoradiation increased the percentage of sarcopenia. Sarcopenic patients are at higher risk for increased toxicity during neoadjuvant radiochemotherapy and showed a non-significant trend to more postoperative morbidity.

## Background

Sarcopenia, the critical depletion of skeletal muscle mass, is an independent predictor of survival in several tumor entities and can lead to a better risk stratification for treatment-related complications than age and body mass index (BMI) [1–3]. The advantage of sarcopenia as prognostic factor is that it can be easily assessed on cross-sectional computed tomography (CT) imaging which is usually performed in cancer patients for staging [3].

Initial weight loss and nutritional status are known for a long time as adverse prognostic factors for surgical complications and overall survival in esophageal cancer patients [4–6]. In recent years, there have been a rapidly increasing number of publications on the role of sarcopenia in esophageal cancer [7, 8]. Although current meta-analyses suggest an inferior survival for sarcopenic patients with esophageal cancer [7, 8], the results of the individual patient series are conflicting, most likely due to the heterogeneity of the investigated patient populations. While sarcopenic patients had an inferior outcome in some series [9, 10], this correlation was not observed in more selected groups such as patients receiving trimodality therapy [11, 12] or in patients younger than 65 years [13]. However, there is still a lack of data on the role of sarcopenia in homogeneous patient groups which have been selected by stringent study inclusion criteria.

Likewise, it is currently controversial whether sarcopenia is a risk factor for increased adverse events during neoadjuvant treatment and surgery [7]. Most previous reports have focused on postoperative morbidity and mortality, and data on the role of sarcopenia specifically in patients receiving trimodality treatment is scarce [12, 14]. Finally, it is also debated whether previously published cut-off values for sarcopenia are applicable to all cancer patient populations [7].

The aim of our study was therefore to assess the impact of sarcopenia in patients with locally advanced esophageal cancer treated with induction chemotherapy, neoadjuvant radiochemotherapy and surgery with or without cetuximab within the phase III trial SAKK 75/08 [15].

## Methods

The intergroup phase III trial SAKK 75/08 randomized 300 patients with locally advanced esophageal cancer to receive trimodality therapy with or without cetuximab at 53 European centers between May 2010 and December 2013. Trimodality therapy consisted of induction chemotherapy (two cycles of docetaxel 75 mg/m^2^, cisplatin 75 mg/m^2^) and neoadjuvant radiochemotherapy (45 Gy; docetaxel 20 mg/m^2^ and cisplatin 25 mg/m^2^ weekly for 5 weeks) with or without cetuximab (250 mg/m^2^ weekly). In the experimental arm, cetuximab (500 mg/m^2^ every 2 weeks) was continued postoperatively for 12 weeks as adjuvant treatment [15]. Eligibility criteria were patients with resectable esophageal squamous cell or adenocarcinoma (including Siewert II) with the Union for International Cancer Control (UICC) TNM stages cT2+, cT3 cNx or cT4a cNx, 18–75 years old and with an Eastern Co-operative Oncology Group (ECOG) performance status of 0–1. Cervical tumors and tumors within the first 5 cm of the thoracic esophagus were not eligible. Radiotherapy was delivered in 25 daily fractions of 1.8 Gy to a total dose of 45 Gy with weekly concomitant chemotherapy. The clinical target volume (CTV) was defined by anatomically adapted margins around the macroscopic tumor of 3.5 cm longitudinally and 1 cm radially. Additionally, all positive nodes were covered and the coeliac nodes were included in the CTV for distal esophageal cancers. Planning treatment volume (PTV) was 1 cm. Median time between the end of radiotherapy and surgery were 6 weeks (range: 3–18 weeks). The primary endpoint was progression-free survival (PFS), secondary endpoints were overall survival (OS), time to locoregional and distant failure after R0 resection, postoperative complications, in-hospital mortality, the rate of R0 resection and pathological remission.

With a median follow-up of 4 years, the study showed a significant improvement of the time to loco-regional failure after R0-resection by the addition of cetuximab to trimodality therapy (HR 0.53 (0.31–0.90), *p* = 0.017). Additionally, there was a non-significant, but obvious trend towards improved OS in the experimental arm with a median OS of 5.1 versus 3.0 years (HR 0.73 (0.52–1.01), *p* = 0.055). There was no statistically significant improvement of progression-free survival (*p* = 0.13) [15].

For our substudy, only patients from four sites in Switzerland, Kantonsspital St. Gallen, Inselspital Bern, Universitätsspital Zürich and Kantonsspital Winterthur, were included.

Adverse events were collected according to the Common Terminology Criteria for Adverse Events (CTCAE) version 4.0.

### CT-based assessment of skeletal muscle mass

**Fig. 1:**
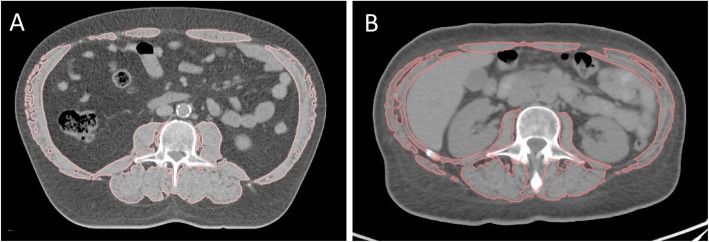
Cross-sectional abdominal CT on the level of the third lumbal vertebra. Skeletal muscle surface is delineated in a non-sarcopenic patient (**a**) and in a sarcopenic patient (**b**)

Skeletal muscle surface on cross-sectional CT images has been demonstrated to correlate well with total body muscle mass [16] and is considered the gold standard for body composition analysis in cancer patients [3]. A single cross-sectional abdominal CT slice at the height of the third lumbar vertebra (L3) was used as representative for the whole body composition (Fig. 1) [17, 18]. The following CT scans were used for the analysis: CT from the initial staging PET-CT before induction chemotherapy (per protocol within 6 weeks before the start of neoadjuvant therapy); radiotherapy planning CT after induction chemo (immuno) therapy and before radiochemo (immuno) therapy (RC(I)T, week six of neoadjuvant therapy); re-staging CT after radiotherapy and before surgery (week 12–15 of neoadjuvant therapy).

Image analysis of anonymized CT slices was performed with a dedicated research software (Slice-O-Matic™, Tomovision, Magog, Canada) as described previously [19].

Skeletal muscle was identified anatomically and quantified using Hounsfield unit (HU) thresholds (− 29 to + 150) [20] and contains at the L3 level psoas, paraspinal muscles (erector spinae, quadratus lumborum), and abdominal wall muscles (transversus abdominus, external and internal obliques, rectus abdominus) [21]. The cross-sectional muscle area at this height (cm^2^) was normalised for stature (L3 skeletal muscle index, cm^2^/m^2^), as it is linearly related to whole-body muscle mass [16]. Additionally, data on cross-sectional total adipose tissue was obtained as previously described [22]. Sarcopenia was defined by previously published sex-specific cut-offs for L3 skeletal muscle index (men: 43 m^2^/m^2^ for body mass index (BMI) < 25 kg/m^2^, 53 cm^2^/m^2^ for BMI ≥ 25 kg/m^2^; women: 41 cm^2^/m^2^) [21]. Additionally, we performed in 57 male patients a receiver operating characteristics (ROC) curve analysis in order to investigate the additional value of population-specific cut-off values for sarcopenia.

### Statistical analysis

Sarcopenia was assessed for patients from the aforementioned institutions in Switzerland. A total of 61 patients had sarcopenia assessment for at least two time points. Baseline sarcopenia was available for 60 patients. The loss of muscle mass between baseline and surgery was calculated as the difference in skeletal muscle index between start of induction chemo (immuno) therapy and surgery.

Sarcopenia endpoints were summarized descriptively using median and range for continuous data and frequency and percentage for categorical data overall as well as separately by treatment arm.

Continuous data was summarized using median and range and compared between subgroups using Wilcoxon rank-sum tests. Categorical data was summarized using frequency counts and percentages and compared between subgroups using Fisher’s exact tests. Time-to-event endpoints were summarized by the median and corresponding 95% confidence interval (CI) using the Kaplan-Meier method. The number and type of events were presented descriptively by frequency and percentage. Comparisons of time-to-event endpoints between subgroups were performed using the log-rank test.

All analyses were performed using SAS 9.4 and R 3.4.3.

## Results

### Analysis using standard cut-off values for sarcopenia

Sixty-one patients from four centers in Switzerland were included in this substudy and showed similar patient characteristics and tumor stage and histology compared to the whole SAKK 75/08 study population (Table 1) [15].

**Table 1:** Patient characteristics in sarcopenia substudy and whole SAKK 75/08 population

	SAKK 75/08 whole population	Sarcopenia substudy population
n	300	61
Age (years; median and range)	61 (36, 75)	61 (38, 75)
Sex		
Female	37 (12.3%)	4 (6.6%)
Male	263 (87.7%)	57 (93.4%)
Clinical stage		
T2 N+	44 (14.7%)	16 (26.2%)
T3 N0	31 (10.3%)	2 (3.3%)
T3/4 N+	225 (75.0%)	43 (70.5%)
Performance Status		
0	189 (63.0%)	32 (52.5%)
1	109 (36.3%)	29 (47.5%)
Histologic Type		
AC	189 (63.0%)	50 (82.0%)
SCC	111 (37.0%)	11 (18.0%)

*AC* Adenocarcinoma, *SCC* Squamous cell carcinoma. Baseline L3 muscle index was not assessable in one patient

Skeletal muscle surface derived from CT imaging was available for 98% of the included patients at baseline staging, for 89% before neoadjuvant RC(I)T and for 100% after neoadjuvant treatment before surgery.

Sarcopenia defined by published cut-offs [21] was found at baseline in 29.5% of the included patients. We found no significant differences between the group of sarcopenic and non-sarcopenic patients regarding body mass index, age, performance status, histologic type, T stage and N stage.

Over the course of neoadjuvant treatment there was a significant reduction of the L3 muscle index from a median of 52.2 to 46.5 cm^2^/m^2^ (*p* < 0.0001). Also, total adipose tissue was significantly reduced over the neoadjuvant treatment course (*p* = 0.041). The prevalence of sarcopenia was similar in both treatment arms of the study at the initial radiological staging (*p* = 1.0) and progressed significantly under neoadjuvant therapy from 29.5 to 63.9% in the whole investigated population (*p* < 0.0001, see Table 2).

**Table 2:** Sarcopenia endpoints (*n* = 61)

Variable	Before induction therapy (baseline)	Before RC(I)T	Before surgery	Change between baseline and surgery (*p*-value*)
Skeletal muscle surface (cm^2^)	160.1 (72.8, 211.4)	144.9 (66.5, 198.0)	144.4 (61.4, 187.0)	−14.6 (*p* < 0.0001)
Total adipose tissue (cm^2^)	294.9 (24.1, 823.6)	292.4 (29.9, 841.1)	285.7 (64.1, 756.6)	−10.7 (*p* = 0.041)
L3 skeletal muscle index (cm^2^/ m^2^)	52.2 (26.7, 75.8)	47.3 (24.4, 66.9)	46.5 (22.6, 67.1)	−5.0 (*p* < 0.0001)
Sarcopenia	29.5%	49.2%	63.9%	+ 34.4% (*p* < 0.0001)

*RC(I)T* Radiochemo (immuno) therapy. Values are depicted as median (range). Sarcopenia was defined according to established sex-specific cut-off values [21]. *Mc Nemar test for categorical variables and Wilcoxon rank-sum test for continuous variables

### Association of sarcopenia with adverse events during neoadjuvant therapy and surgery

Sarcopenia at baseline according to published cut-offs from Martin et al. [21] showed a significantly higher rate of grade ≥ 3 adverse events during RC(I)T and a non-significant trend to higher postoperative morbidity (Table 3). We observed no difference in toxicity during induction chemotherapy in sarcopenic and non-sarcopenic patients. The only two cases of postoperative death occurred in the non-sarcopenic patient group. Sarcopenia before RC(I)T assessed at the radiotherapy planning CT showed a significantly increased grade ≥ 3 toxicity during RC(I)T (80.0% vs. 34.8%, *p* = 0.002). Sarcopenia assessed directly before surgery showed a numerical, but non-significant difference in surgical complications (56.4% vs. 36.4%, *p* = 0.18).

**Table 3:** Adverse events and surgical complications by sarcopenia at baseline

Variable	No sarcopenia (*N* = 29)	Sarcopenia (*N* = 31)	
	n (%)	n (%)	*p*-value
AE grade ≥ 3 during induction chemotherapy			0.78
No	22 (52.4%)	8 (44.4%)	
Yes	20 (47.6%)	10 (55.6%)	
AE grade ≥ 3 during RC(I)T			0.041
No	20 (47.6%)	3 (16.7%)	
Yes	22 (52.4%)	15 (83.3%)	
Overall surgical complications			0.16
No	24 (57.1%)	6 (33.3%)	
Yes	18 (42.9%)	12 (66.7%)	
Hospitalization during neoadjuvant therapy			1
No	5 (11.9%)	2 (11.1%)	
Yes	37 (88.1%)	16 (88.9%)	
Duration of postoperative hospitalization			
Median (range)	18.5 (10.0, 144.0)	20.5 (10.0, 49.0)	0.43

*AE* Adverse event, *RC(I)T* Radiochemo (immune) therapy

### Feasibility of therapy

Neoadjuvant therapy and surgery were feasible both in sarcopenic and non-sarcopenic patients in the majority of patients without statistically significant difference between these groups: all patients completed induction chemotherapy and 92% neoadjuvant radiochemotherapy. Surgery was performed in 97%. The frequency of hospital admissions during neoadjuvant therapy was not significantly higher in sarcopenic patients. We observed no significant difference in postoperative hospitalization in patients with sarcopenia at baseline (median days 20.5 vs. 18.5, *p* = 0.43) and when sarcopenia was assessed directly before surgery (20 vs. 19.5 days, *p* = 0.69). Also, there was no significant difference in time spent on the intensive care unit postoperatively between sarcopenic and non-sarcopenic patients defined at baseline (*p* = 0.58).

### Impact of sarcopenia on oncological outcome

**Fig. 2:**
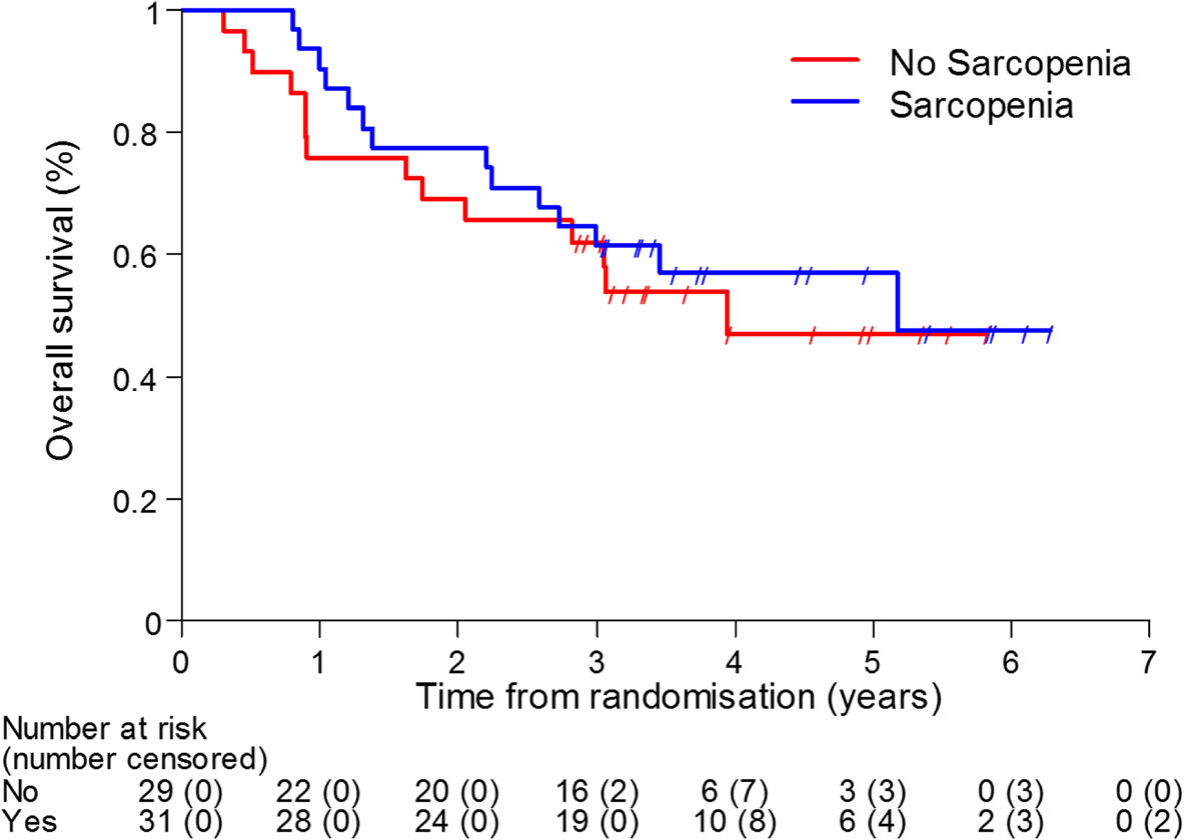
Kaplan-Meier plot for OS by sarcopenia at baseline (*p* = 0.72)

We observed no significant difference in progression-free survival (PFS, *p* = 0.44) and overall survival (OS, *p* = 0.72, see Fig. 2) between sarcopenic and non-sarcopenic patients when the muscle index at baseline was considered.

Using population-specific cut-offs for sarcopenia defined by ROC analysis gave similar results compared to the standard cut-offs by Martin et al. [21]: Sarcopenia defined by a L3 skeletal muscle index of 47.5 cm^2^/m^2^ or lower before RC(I)T resulted in a significantly higher rate of grade ≥ 3 adverse events during RC(I)T (78.3% vs. 38.5%; *p* = 0.009). There was no significant correlation between sarcopenia defined by the population-specific cutoff at baseline and OS (*p* = 0.57).

## Discussion

There is a growing body of literature on the impact of low skeletal muscle mass, i.e. sarcopenia on toxicity and outcome in patients treated for esophageal cancer [7]. Several retrospective patient series have been published with inconsistent results regarding the correlation of sarcopenia and impaired survival as well as increased adverse events [7, 8].

To our knowledge, our study is the first association of skeletal muscle mass with prospectively collected CTCAE adverse events and survival data in patients undergoing curatively intended therapy for locally advanced esophageal cancer. We investigated the role of sarcopenia in a selected, homogeneous patient population with good performance status and no severe comorbidities which met the inclusion criteria of the SAKK 75/08 phase III trial.

During the course of neoadjuvant therapy we observed a statistically significant loss of muscle mass and consequently increased percentage of sarcopenic patients in the whole study population. These findings have also been described in previous retrospective studies and have been associated with inferior survival [23, 24].

In our substudy of the phase III trial SAKK 75/08, we found significantly increased adverse events during RC(I)T and a non-significant trend for increased surgical morbidity in sarcopenic patients. However, sarcopenic patients showed the same treatment compliance including surgery as non-sarcopenic patients and showed no increased postoperative mortality. Due to small sample size and exploratory nature of our analysis, correlation of long-term outcome regarding survival should be interpreted with caution.

A recent meta-analysis of several previously published retrospective series found a negative impact of sarcopenia on survival in patients with esophageal cancer [7]. There was no significant association of sarcopenia with postoperative morbidity except for pulmonary infections [7]. Most of the previous published series reporting increased toxicity or mortality for sarcopenic patients were retrospective in nature and included a heterogeneous population of patients with non-metastatic esophageal cancer. In contrast, our population met concise study inclusion criteria and was able to undergo trimodality therapy [15]. This is a significant selection bias compared to the general population of patients with locally advanced esophageal cancer. We cannot exclude that sarcopenia served in previously published patient series as a surrogate parameter for poor general condition and advanced tumor stage and may therefore have a more pronounced effect on oncologic outcome than in our study. This hypothesis is supported by other studies which did not find a significantly inferior survival in sarcopenic esophageal cancer patients which were able to undergo esophagectomy [10, 11, 14].

There is only limited data on the role of sarcopenia regarding neoadjuvant radiochemotherapy. Our results showing increased toxicity during neoadjuvant radiochemotherapy in sarcopenic patients have to be highlighted as most studies were mainly focused on postoperative morbidity and survival [7]. In line with our findings, a retrospective series by Murimwa et al. sarcopenic patients identified by the less commonly used L4 psoas muscle index showed increased acute toxicity without negative impact on survival [12]. However, several publications question the validity of psoas muscle index [25, 26].

Our study had limitations. First, data were only available for a subgroup of the SAKK 75/08 trial at four sites in Switzerland, but patient characteristics were similar to the whole study population. However, our study may be underpowered to detect small differences between the group of sarcopenic and non-sarcopenic patients, particularly regarding outcome. Our analysis might be biased by different intervals between the three time points of skeletal muscle mass assessment: The last CT-based measurement was done directly before surgery with a median interval of 6 weeks after the end of RC(I)T, but 19% of the patients had an interval longer than 7 weeks. Additionally, it is possible that our study underestimates the actual loss of muscle mass during RC(I)T, as patients had time to recover in the interval until surgery. Also, patients from both treatment arms, i.e. patients receiving trimodality treatment with or without cetuximab, were included. The prevalence of sarcopenia was similar in both treatment arms in our substudy, nevertheless we cannot exclude any interaction of cetuximab with sarcopenia. While the SAKK 75/08 control arm is standard in many Swiss centers, neoadjuvant RCT for esophageal cancer is more commonly performed according to the less intense CROSS protocol [27]. It is not clear whether the increased toxicity of neoadjuvant RCT in sarcopenic patients is as pronounced as in our study when the CROSS regimen is used (41.4 Gy in 23 fractions and weekly chemotherapy with carboplatin and paclitaxel without induction chemotherapy) [27]. Finally, we were not able to perform separate analyses for adenocarcinoma and squamous cell carcinoma of the esophagus due to the limited patient number.

In conclusion, our findings show that trimodality therapy in locally advanced esophageal cancer according to the SAKK 75/08 protocol is also feasible in sarcopenic patients despite higher grade 3–4 toxicity during neoadjuvant RC(I)T. The role of sarcopenia in esophageal cancer needs to be further investigated in order to improve the identification of patients at risk for increased toxicity, as these patients may benefit from more intensive monitoring and supportive therapy.

## Data Availability

The datasets used and analyzed during the current study are available from the corresponding author on reasonable request.

## Abbreviations

BMI: Body mass index
CT: Computed tomography
CTCAE: Common Terminology Criteria for Adverse Events
CTV: Clinical target volume
ECOG: Eastern Co-operative Oncology Group
HU: Houndsfield units
PTV: Planning target volume
RC(I)T: Radiochemo (immuno) therapy
SMI: Skeletal muscle index
UICC: Union for International Cancer Control
